# Treadmill Exercise Prevents Decline in Spatial Learning and Memory in 3×Tg-AD Mice through Enhancement of Structural Synaptic Plasticity of the Hippocampus and Prefrontal Cortex

**DOI:** 10.3390/cells11020244

**Published:** 2022-01-12

**Authors:** Lianwei Mu, Jiajia Cai, Boya Gu, Laikang Yu, Cui Li, Qing-Song Liu, Li Zhao

**Affiliations:** 1 Key Laboratory of Physical Fitness and Exercise, Ministry of Education, Beijing Sport University, Beijing 100084, China; lmu@mcw.edu (L.M.); caijj_2008@163.com (J.C.); guboya@sohu.com (B.G.); yulaikang@126.com (L.Y.); 13121681899@163.com (C.L.); 2 Department of Pharmacology and Toxicology, Medical College of Wisconsin, 8701 Watertown Plank Road, Milwaukee, WI 53226, USA; qsliu@mcw.edu; 3 School of Physical Education (Main Campus), Zhengzhou University, Zhengzhou 450001, China

**Keywords:** exercise, memory, synaptic, dendritic spines, structural synaptic plasticity, 3×Tg-AD mice

## Abstract

Alzheimer’s disease (AD) is characterized by deficits in learning and memory. A pathological feature of AD is the alterations in the number and size of synapses, axon length, dendritic complexity, and dendritic spine numbers in the hippocampus and prefrontal cortex. Treadmill exercise can enhance synaptic plasticity in mouse or rat models of stroke, ischemia, and dementia. The aim of this study was to examine the effects of treadmill exercise on learning and memory, and structural synaptic plasticity in 3×Tg-AD mice, a mouse model of AD. Here, we show that 12 weeks treadmill exercise beginning in three-month-old mice improves spatial working memory in six-month-old 3×Tg-AD mice, while non-exercise six-month-old 3×Tg-AD mice exhibited impaired spatial working memory. To investigate potential mechanisms for the treadmill exercise-induced improvement of spatial learning and memory, we examined structural synaptic plasticity in the hippocampus and prefrontal cortex of six-month-old 3×Tg-AD mice that had undergone 12 weeks of treadmill exercise. We found that treadmill exercise led to increases in synapse numbers, synaptic structural parameters, the expression of synaptophysin (Syn, a presynaptic marker), the axon length, dendritic complexity, and the number of dendritic spines in 3×Tg-AD mice and restored these parameters to similar levels of non-Tg control mice without treadmill exercise. In addition, treadmill exercise also improved these parameters in non-Tg control mice. Strengthening structural synaptic plasticity may represent a potential mechanism by which treadmill exercise prevents decline in spatial learning and memory and synapse loss in 3×Tg-AD mice.

## 1. Introduction

Alzheimer’s disease (AD) is a progressive neurodegenerative disease most often characterized by memory impairment and cognitive decline [1]. Difficulty in remembering newly learned information, or short-term memory loss, is an early symptom of AD [2]. As the disease progresses, AD patients gradually experience long-term memory loss, which severely impacts the quality of life. AD pathology is characterized by the accumulation of amyloid-β (Aβ) plaques and tau neurofibrillary tangles in the hippocampus and the prefrontal cortex (PFC), both of which are key structures in learning and memory [3,4,5,6]. Previous studies suggest that Aβ and tau can impair memory by disrupting synaptic plasticity in the hippocampus and prefrontal cortex [7,8]. This synaptic plasticity includes activity-dependent changes in the synaptic efficacy and remodeling, axonal sprouting and dendritic remodeling, and dendritic spine dynamics. In animal studies, soluble Aβ-oligomers at nanomolar concentrations, extracted directly from the cerebral cortex of typical AD patients or generated from synthetic peptide, disrupted the memory of a learned behavior by potently inhibiting long term potentiation (LTP), enhancing long term depression (LTD), reducing dendritic spine density of hippocampus [9,10,11]. Meanwhile, it has been shown that spatial learning and memory impairment is caused by synaptic dysfunction both in human patients and different transgenic mouse models of AD, e.g., App^NL-G-F^ [12,13], Tg2576 [14], APP/PS1 [15], 5×FAD [16], and 3×Tg-AD [17]. The reduced synaptic function was often observed in young mice prior to plaque development, which indicated that Aβ oligomers trigger synaptic deficits prior to plaque formation [18]. Meanwhile, studies using computed tomography (CT) and magnetic resonance imaging (MRI) show that the hippocampal and prefrontal cortex of people with Alzheimer’s disease shrink dramatically as the disease progresses [19,20]. AD is increasingly prevalent around the world, and yet no available treatments can cure or slow the progression of this disease.

Accumulating evidence suggests that physical exercise decreases β amyloid burden and improves synaptic plasticity and learning and memory deficits in rodent models of AD and delays the progress of pathological changes in the brain [21,22,23,24,25,26,27,28]. Physical exercise may act through various mechanisms in different experimental models. Aerobic exercise can increase cerebral blood flow, improve oxygen utilization, and upregulate the expression of growth factors that promote synaptic plasticity and neurogenesis, and can exert a direct beneficial effect on brain function [29,30,31]. Mechanisms involved include the upregulation of gene expression of molecular targets associated with learning and memory, synaptic plasticity, and neuronal survival [30]. Furthermore, aerobic exercise could ameliorate learning and memory deficits by reversing synapse loss in the hippocampus and cortex in aging rats, a rat model of vascular dementia, and a middle cerebral artery occlusion/reperfusion (MCAO/R) model [31,32,33]. The present study will examine the effects of 12 weeks of treadmill exercise on spatial learning and memory using an eight-arm radial maze in six-month-old 3×Tg-AD mice. Then, the potential mechanisms involved are investigated. The hippocampus and prefrontal cortex are known to be critical brain regions for spatial learning and memory [34]. It is believed that synaptic plasticity within the hippocampus and prefrontal cortex is the cellular basis of learning and memory, which depends on the strengthening of synaptic connections [35]. Previous studies have uncovered that the larger synaptic active zone is more effective at exciting postsynaptic neurons and shortening of the active zone may reflect a condition of impaired efficiency of synaptic transmission [36]. The neurotransmitter diffuses across the synaptic cleft and binds to receptor proteins on the membrane of the postsynaptic neuronal, causing ionic channels in the postsynaptic membrane to either open or close. The optimal shortening of the synaptic cleft extent and thickening of the postsynaptic density (PSD) can enhance synaptic transmission efficiency [37,38,39]. In addition, axon, dendritic, and dendritic spines constitute the structural basis of structural synaptic plasticity. In the present study, we determined whether 12 weeks of treadmill exercise pretreatment altered Aβ-induced impairment in structural synaptic plasticity within the hippocampus and prefrontal cortex in six-month-old 3×Tg-AD mice. We found that 12 weeks of treadmill exercise prevented a decline in spatial learning and memory in six-month-old 3×Tg-AD mice and that treadmill exercise led to enhanced structural synaptic plasticity by increasing synapse numbers and synaptic structural parameters, the expression of synaptophysin (Syn), the axon length, dendritic complexity, and the dendritic spine numbers. These mechanisms may underlie the treadmill exercise-induced improvement of learning and memory.

## 2. Materials and Methods

### 2.1. Animals

Triple-transgenic Alzheimer’s disease (3×Tg-AD) male mice (harboring PS1M146V knockin, APPswe and TauP301L transgenes) were acquired from The Jackson Laboratory. Age-matched C57BL/6J male mice were employed as non-transgenic (Non-Tg) controls. The study was performed on 3-month-old 3×Tg-AD (n = 20) and non-Tg mice (n = 20). All animals were given ad libitum access to food and water in a room maintained at controlled temperature (23 ± 1 °C) and humidity (40–60%), with a 12 h light-dark cycle. Mice were handled daily for 3–6 days prior to behavior experiments. All animal maintenance and use were in accordance with protocols approved by the ethical committee of Beijing Sport University (2015015).

### 2.2. Treadmill Exercise Protocol

**Figure 1:**
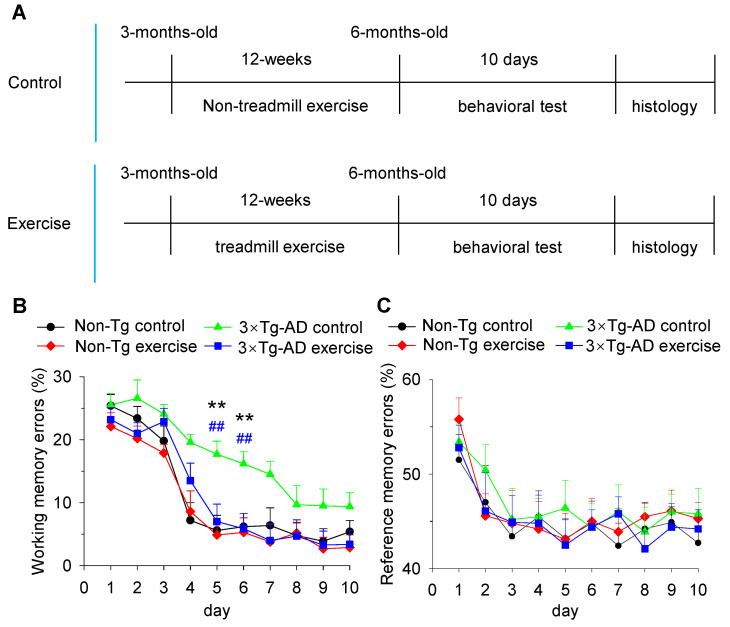
Treadmill exercise prevents decline in spatial learning and memory in 3×Tg-AD mice. (**A**) Timeline of treadmill exercise or non-exercise control, behavioral test and histological test. (**B**) The percentage of working memory errors on day 5 and day 6 was significantly increased in the 3×Tg-AD mice compared to the Non-Tg control group (**B**; ** *p* < 0.01, n = 10 mice). The increase in the percentage of working memory errors was prevented by treadmill exercise pretreatments (**B**; ## *p* < 0.01, n = 10 mice). (**C**) There were no significant effects on the percentage of reference memory errors between Non-Tg control, Non-Tg exercise, 3×Tg-AD control, and 3×Tg-AD exercise mice (**C**; *p* > 0.05, n = 10 mice).

At 3 months of age, mice were randomly assigned into four groups (n = 10 each): Non-Tg control group, Non-Tg exercise group, 3×Tg-AD control group, 3×Tg-AD exercise group. All of the exercised mice were allowed to adapt to treadmill running for 30 min on 3 consecutive days (first day at 5 m/min; second and third day at 10 m/min). Then, those mice were subjected to a treadmill exercise protocol at the speed of 12 m/min on a 0° slope for 10 min, then 15 m/min for 50 min. In total, mice were trained for 1 h per day, 5 days per week, for a total of 12 weeks. According to our test by an enclosed single-lane treadmill attached to an Oxymax/Comprehensive Laboratory Animal Monitoring System, this long-duration training protocol maintains exercise intensity from 50% to 65% of VO2 max. Mice in the non-Tg control and 3×Tg-AD control groups were left on the treadmill, without running, for the same period as exercise groups. We first carried out the eight-arm radial maze test for all mice (10 mice in each group, 6 months of age). Next, we examined the histological abnormalities by Golgi staining (3 mice in each group), Western blot (3 mice in each group), and electron microscopy (4 mice in each group) (Figure 1A).

### 2.3. Eight-Arm Radial Maze Test

The eight-arm radial maze consisted of a central platform with a diameter of 18 cm and eight arms that extended from the central platform, each 30 cm long, 7 cm wide, and 15 cm high, with an equal angular distance between adjacent arms. The eight-arm radial maze was painted blue. The end of each arm held a food cup that was 3 cm in diameter and 1 cm deep. A door was located between each arm and the center of the maze. A video camera located above the maze was connected to a computer to record the movement of the animals. The maze was elevated 35 cm above the floor level in a dimly lit room with several extra-maze cues. The whole experimental process used chocolate as food. The data were analyzed with the ethology video analysis software (JLBehv-8ARMM, Shanghai Jiliang Company, Shanghai, China).

To enhance motivation for chocolate in the radial maze protocol, mice were food-restricted during an additional week to reach 80–85% of the original body weight and maintained under these conditions during the entire study. The radial maze task was divided into adaptation and acquisition sessions. On the two consecutive days of the adaptation session, each mouse was placed in the central starting platform. After 10 s, all doors were opened, and the mouse was allowed to explore and consume food (0.08 g chocolate) in each arm of the maze for 10-min. The acquisition session consisted of one trial performed once a day for 10 consecutive days. Four of the arms (1, 3, 5, and 7) were baited with chocolate rewards. Mice were placed in the center of the maze, and after 10 s, all doors were opened. The activities of mice were recorded automatically and then analyzed using a video tracking system. An arm entry was counted when all four paws of the mouse crossed the start of the arm. The trial continued until the mouse entered all four baited arms. At the end of the trial, the mouse was returned to the home cage. Entry in a non-baited arm was considered as a reference memory error and a re-entry in a previously visited baited arm was considered as a working memory error. In tabulating the performance, the percentage of working memory errors (short-term memory) was defined as any repeated entries among total entries, and the percentage of reference memory errors (long-term memory) as the percentage of entries into an unbaited arm and those without consumption of bait when entering baited arms out of total entries [40,41].

### 2.4. Golgi Staining

For the quantification of morphological changes, the hippocampus and prefrontal cortex were stained with a Golgi staining kit (FD Rapid Golgi Stain Kit PK-401, FD Neuro Technologies, Columbia, MD, USA) following the manufacturer’s instructions. In brief, brains were extracted and placed in jars containing 20 mL of Golgi-Cox solution for 17 days in the dark before being cut into 60 µm slices using a cryostat (Leica, Nussloch, Germany). The sections were incubated in the staining solution for 10 min, dehydrated in an ascending ethanol series (50%, 70%, 95%, and 100%), and cleared in xylene two times. Finally, slides were cover-slipped with Permount.

Pyramidal cells from the CA1 region of the hippocampus or the layer V of the prefrontal cortex were drawn using the microscope at a magnification of 200× and 1000×. Neurons were selected if they were un-obscured (by vasculature, glial cells, or other neurons) and appeared to be wholly stained and intact within the section. The length of axon was measured by manually tracing a neurite from the boundary of the soma to the tip of the axon [42]. Dendritic morphology was assessed by Sholl analysis. A transparent grid with equidistant (10 μm) concentric rings was centered over the dendritic tree tracings. The number of ring intersections was used to estimate the total dendritic length and dendritic arborization [43,44,45]. Finally, we collected 9–13 sections in the CA1 hippocampus and layer V prefrontal cortex from 3 mice in each experiment group. To calculate the spine number, a length of dendrite (at least ≥10 μm long, 2nd-oder dendrites) was traced (at 1000×). The exact length of each dendritic branch was calculated, and the number of spines along the length counted (to yield spines/10 μm) [43]. The length of dendritic filopodia is normally >3 μm and <10 μm [46]. The morphology of spines is commonly categorized into three types: thin, mushroom, and stubby [47]. Spines are considered thin if the length is greater than the neck diameter and the diameters of the head and neck are similar. Spines are classified as mushrooms if the diameter of the head is greater than the diameter of the neck. Spines are considered stubby if the length and width are equal.

### 2.5. Western Blot

Mice were anesthetized by isoflurane inhalation and decapitated. Tissues of hippocampus or prefrontal cortex were lysed in cold RIPA lysis buffer (Thermo Scientific Pierce, Waltham, MA, USA) supplemented with protease and phosphatase inhibitor cocktail (Roche, Indianapolis, IN, USA). Total protein was measured using the BCA assay (Thermo Scientific Pierce, USA). Samples were heated at 100 °C for 15 min. For Western blot, equal amounts of protein were loaded (20 ug/well) onto a 12% gel. After electrophoresis and transfer to PVDF membranes (Millipore, IPVH00010, Burlington, MA, USA), they were incubated for 1 h at room temperature with a blocking buffer (5% BSA buffer). The PVDF membranes were incubated overnight with the primary antibody (GAPDH, 1:10,000, Abcam, Boston, MA, USA; PSD95, 1:500, Abcam, USA; Syn, 1:500, Abcam, USA). They were then washed with a TBST buffer three times at 5 min intervals and incubated for 1 h with an HRP-conjugated goat anti-rabbit or HRP-conjugated goat anti-mouse secondary antibody (1:15,000, Proteintech Group, Rosemont, IL, USA), followed by TBST washes at 5 min intervals. Protein levels were detected with chemiluminescent reagents (Thermo Scientific Pierce, Waltham, MA, USA) and the bands were measured using Image J software.

### 2.6. Electron Microscopy

**Figure 2:**
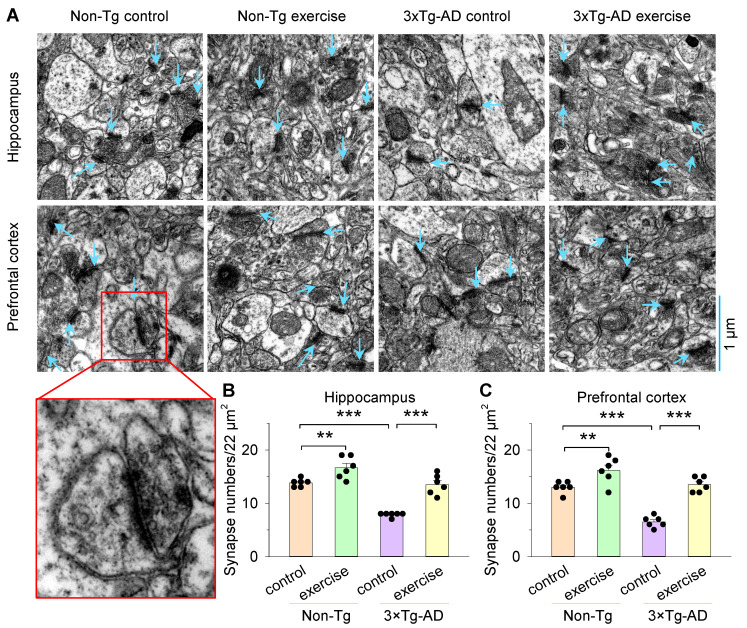
Treadmill exercise increases synapse numbers of hippocampus and prefrontal cortex in 3×Tg-AD mice. (**A**) Representative electron microscope imaging of hippocampus and prefrontal cortex in non-Tg control, non-Tg exercise, 3×Tg-AD control, and 3×Tg-AD exercise mice. The synapses are marked by the blue arrowheads. Red box represents an enlarged synapse. An expanded, high magnification view of synapses in the prefrontal cortex is shown in the red square box in the bottom. (**B**,**C**) The synapse numbers of hippocampus (**B**) and prefrontal cortex (**C**) were significantly decreased in the 3×Tg-AD control group compared to the non-Tg control group (*** *p* < 0.001, n = 6 image sections), and this decrease was blocked by treadmill exercise pretreatment both in the hippocampus (**B**) and prefrontal cortex (**C**) (*** *p* < 0.001, n = 6 image sections). Treadmill exercise pretreatment increased the synapse numbers of the hippocampus (**B**) and prefrontal cortex (**C**) in non-Tg mice (** *p* < 0.01, n = 6 image sections). Each data set was obtained from 4 mice.

**Figure 3:**
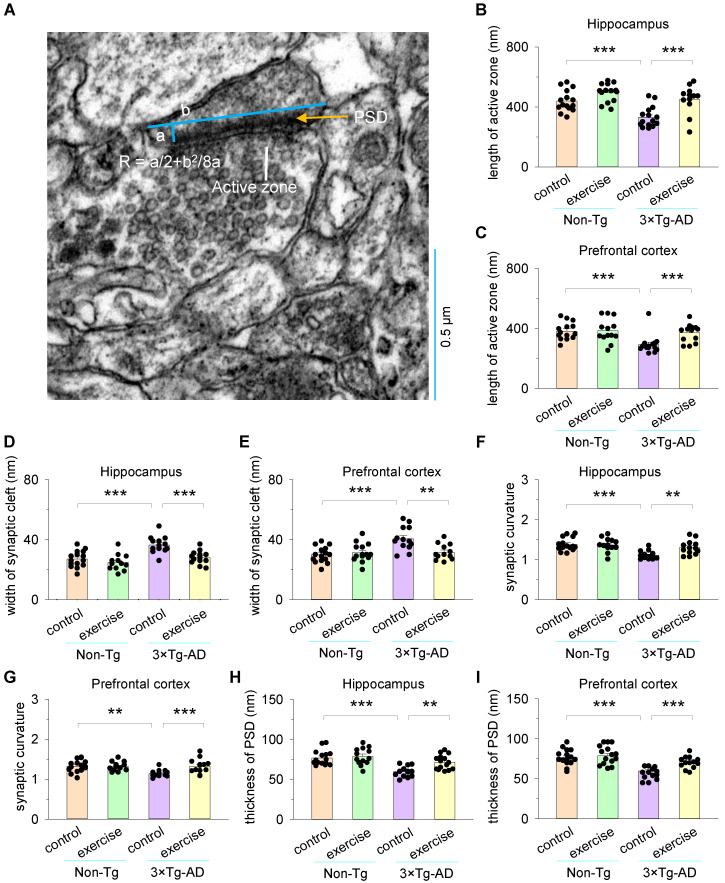
Treadmill exercise improves synaptic structural parameters of the hippocampus and prefrontal cortex in 3×Tg-AD mice. (**A**) A representative measurement of synaptic structural parameters. The formula R = a/2 + b^2^/8a, where b is the line joining the two ends of the postsynaptic thickening and a is the perpendicular distance from the postsynaptic membrane to b, was used to determine synaptic curvature. (**B**,**C**) The length of the synaptic active zone of hippocampus (**B**) and prefrontal cortex (**C**) was significantly decreased in the 3×Tg-AD control group compared to the non-Tg control group (*** *p* < 0.001, n = 12–15 synapses), and this decrease was blocked by treadmill exercise pretreatment both in the hippocampus and prefrontal cortex (*** *p* < 0.001, n = 12–15). (**D**,**E**) The width of the synaptic cleft of the hippocampus (**D**) and prefrontal cortex (**E**) was significantly increased in the 3×Tg-AD control group compared to the non-Tg control group (*** *p* < 0.001, n = 12–15 synapses), and this increase was blocked by treadmill exercise pretreatment both in the hippocampus (**D**; *** *p* < 0.001, n = 12–15 synapses) and prefrontal cortex (**E**; ** *p* < 0.01, n = 12–15). (**F**,**G**) The synaptic curvature of the hippocampus (**F**; *** *p* < 0.001, n = 12–15), and prefrontal cortex (**G**; ** *p* < 0.01, n = 12–15) was significantly decreased in the 3×Tg-AD control group compared to the non-Tg control group, and this decrease was blocked by treadmill exercise pretreatment both in the hippocampus (**F**; ** *p* < 0.01, n = 12–15) and prefrontal cortex (**G**; *** *p* < 0.001, n = 12–15). (**H**,**I**) The thickness of postsynaptic density of hippocampus (**H**) and prefrontal cortex (**I**) was significantly decreased in the 3×Tg-AD control group compared to the non-Tg control group (*** *p* < 0.001, n = 12–15), and this decrease was blocked by treadmill exercise pretreatment both in the hippocampus (**H**; ** *p* < 0.01, n = 12–15) and prefrontal cortex (**I**; *** *p* < 0.001, n = 12–15). Each data set consisted of 12 and 15 synapses from 4 mice each group.

Mice were deeply anesthetized with chloral hydrate and perfused transcardially with PBS, followed by 4% paraformaldehyde and 2.5% glutaraldehyde solution in 0.1 M phosphate buffer for 20 min. The brain was removed and immersed in the same fixative overnight. The hippocampus and prefrontal cortex were stained with 1% osmium tetroxide, then dehydrated in a graded series of acetone. Tissues were cut into ultrathin sections, stained with uranyl acetate and lead citrate, and examined with a Hitachi H-7100 electron microscope. Photographs of random positions from these specimens were taken at ×30,000 magnification (22 μm^2^). Synapses with round vesicles, asymmetric synapses, and a single large synaptic contact were presumed to be excitatory synapses, while presynaptic terminals with pleomorphic flattened vesicles and symmetric synapses were presumed to be inhibitory synapses [48]. We analyzed the number of presumable excitatory synapses (22 μm^2^/each image field, Figure 2) in the hippocampus and prefrontal cortex of each mouse, and then averaged the number of synapses of six image fields from the same group (6 image fields from 4 mice in each group). The length of synaptic active zone, width of synaptic cleft, the curvature of synaptic interface, and thickness of post synaptic density from 12–15 randomly selected synapses were quantified and compared from 4 mice per group (Figure 3). The length of active zone and the thickness of postsynaptic density were measured according to Güldner [49]. Synaptic cleft was defined as the brightest region between pre- and postsynaptic membranes [50]. Synaptic curvature was determined using the formula: R = a/2 + b^2^/8a, where b is the line joining the two ends of the postsynaptic thickening and a is the perpendicular distance from the postsynaptic membrane to b [51,52] (see Figure 3A).

### 2.7. Statistics

Data analysis was blind to the genotypes and treatment history of the mice. Data are presented as the mean ± SEM. Data sets were compared with two-way ANOVA followed by Tukey’s *post hoc* analysis. *Post-hoc* analyses were performed only when ANOVA yielded a significant main effect or a significant interaction between the two factors. Results were considered to be significant at *p* < 0.05.

## 3. Results

### 3.1. Treadmill Exercise Prevented Decline in Spatial Learning and Memory in 3×Tg-AD Mice

We first sought to determine whether six-month-old 3×Tg-AD mice exhibited spatial learning and memory impairment and whether treadmill exercise pretreatment prevented decline in spatial learning and memory in six-month-old 3×Tg-AD mice. Non-Tg control mice and 3×Tg-AD mice received 12 weeks of treadmill exercise or non-exercise control treatment beginning at three months of age (2 × 2 factorial design: genotype vs. exercise). After the 12-week training, the eight-arm radial maze test was used to investigate the spatial learning and memory of mice. Both working memory (the ability to remember for a relatively brief period of time) and reference memory (memory for information that is held constant over time) were measured (the difference between working and reference memory has been described in Section 2, Figure 1A). Two-way ANOVA showed that genotype and treadmill exercise had significant effects on the percentage of working memory errors on day 5 (genotype: *F*_1,39_ = 8.6, *p* = 0.006; treadmill exercise: *F*_1,39_ = 5.5, *p* = 0.024; genotype × treadmill exercise interaction: *F*_1,39_ = 4.2, *p* = 0.047; Figure 1B) and day 6 (genotype: *F*_1,39_ = 6.1, *p* = 0.019; treadmill exercise: *F*_1,39_ = 6.4, *p* = 0.016; genotype × treadmill exercise: *F*_1,39_ = 4.6, *p* = 0.039; Figure 1B) of the acquisition session. Tukey’s post hoc tests indicated that the percentage of working memory errors was significantly increased in the 3×Tg-AD control group compared to the non-Tg control group (both day 5 and day 6: *p* < 0.01; Figure 1B). The increase in the percentage of working memory errors was prevented by treadmill exercise pretreatments (both day 5 and day 6: *p* < 0.01; Figure 1B). However, two-way ANOVA found that genotype and treadmill exercise had no significant effects on the percentage of reference memory errors (e.g., day 10, genotype: *F*_1,39_ = 0.2, *p* = 0.6; treadmill exercise: *F*_1,39_ = 0.04, *p* = 0.8; genotype × treadmill exercise interaction: *F*_1,39_ = 0.873, *p* = 0.4; Figure 1C) in all 10 days of the acquisition session. Together, these results suggest that six-month-old 3×Tg-AD mice exhibited impaired spatial working memory but not reference memory, and treadmill exercise prevents a decline in spatial working memory in 3×Tg-AD mice.

### 3.2. Treadmill Exercise Increased Synapse Numbers and Improved Synaptic Structural Parameters of the Hippocampus and Prefrontal Cortex in 3×Tg-AD Mice

To investigate whether treadmill exercise-induced reduction in spatial learning and memory was associated with structural synaptic plasticity, we quantified synapse numbers and synaptic structural parameters of the hippocampus and prefrontal cortex that are critical for the transmission of information related to learning and memory (Figure 2A). Two-way ANOVA revealed that genotype and treadmill exercise had significant effects on the synapse numbers both in the hippocampus (genotype: *F*_1,23_ = 59.3, *p* < 0.001; treadmill exercise: *F*_1,23_ = 51.0, *p* < 0.001; genotype × treadmill exercise interaction: *F*_1,23_ = 5.7, *p* = 0.027; Figure 2B) and prefrontal cortex (genotype: *F*_1,23_ = 48.6, *p* < 0.001; treadmill exercise: *F*_1,23_ = 59.8, *p* < 0.001; genotype × treadmill exercise interaction: *F*_1,23_ = 8.5, *p* = 0.009; Figure 2C). Tukey’s post hoc tests indicated that the synapse numbers of the hippocampus and prefrontal cortex were significantly decreased in the 3×Tg-AD control group compared to the non-Tg control group (*p* < 0.001; Figure 2B,C). Treadmill exercise pretreatment blocked a decrease in the synapse numbers both in the hippocampus and prefrontal cortex in 3×Tg-AD mice (*p* < 0.001; Figure 2B,C). Meanwhile, treadmill exercise increased the synapse numbers in the hippocampus and prefrontal cortex in non-Tg mice (*p* < 0.01; Figure 2B,C).

In order to further assess the efficiency of synaptic transmission, we measured and analyzed the ultra-structural parameters by electron microscopy (EM), including the length of the synaptic active zone, the width of the synaptic cleft, synaptic curvature, and the thickness of the postsynaptic density in the hippocampus and prefrontal cortex (Figure 3A). Previous studies have uncovered that the larger synaptic active zone is more effective at exciting postsynaptic neurons, and the shortening of the active zone may reflect a condition of impaired efficiency of synaptic transmission [36]. Two-way ANOVA showed that genotype and treadmill exercise had significant effects on the length of the synaptic active zone both in the hippocampus (genotype: *F*_1,53_ = 10.6, *p* = 0.002; treadmill exercise: *F*_1,53_ = 5.0, *p* = 0.03; genotype × treadmill exercise interaction: *F*_1,53_ = 9.2, *p* = 0.004; Figure 3B) and prefrontal cortex (genotype: *F*_1,56_ = 17.4, *p* < 0.001; treadmill exercise: *F*_1,56_ = 5.0, *p* = 0.03; genotype × treadmill exercise interaction: *F*_1,56_ = 6.8, *p* = 0.012; Figure 3C). Tukey’s post hoc tests indicated that the length of the synaptic active zone of the hippocampus and prefrontal cortex was significantly decreased in the 3×Tg-AD control group compared to the non-Tg control group (*p* < 0.001; Figure 3B,C). Treadmill exercise pretreatment enhanced the length of the synaptic active zone both in the hippocampus (*p* < 0.001; Figure 3B) and prefrontal cortex (*p* = 0.001; Figure 3C) in 3×Tg-AD mice.

The synaptic cleft is a ~20 nm narrow space between the axon terminal of the presynaptic neuron and the membrane of the postsynaptic neuron. The optimal shortening of the synaptic cleft may have an adaptive function of optimizing synaptic strength by enhancing the effective concentration of released neurotransmitters and decreasing the effective cleft resistance [37,38]. Two-way ANOVA showed that genotype and treadmill exercise had significant effects on the width of the synaptic cleft both in the hippocampus (genotype: *F*_1,55_ = 21.5, *p* < 0.001; treadmill exercise: *F*_1,55_ = 15.1, *p* < 0.001; genotype × treadmill exercise interaction: *F*_1,55_ = 5.4, *p* = 0.025; Figure 3D) and prefrontal cortex (genotype: *F*_1,53_ = 10.6, *p* = 0.002; treadmill exercise: *F*_1,53_ = 5.0, *p* = 0.03; genotype × treadmill exercise interaction: *F*_1,53_ = 9.2, *p* = 0.004; Figure 3E). Tukey’s post hoc tests indicated that the width of the synaptic cleft of the hippocampus and prefrontal cortex was significantly increased in the 3×Tg-AD control group compared to the non-Tg control group (*p* < 0.001; Figure 3D,E). Treadmill exercise pretreatment decreased the width of the synaptic cleft both in the hippocampus (*p* < 0.001; Figure 3D) and prefrontal cortex (*p* = 0.001; Figure 3E) in 3×Tg-AD mice.

Synaptic curvature and the thickness of postsynaptic density are closely correlated with postsynaptic information integration and neurotransmitter transmission efficiency [39]. Two-way ANOVA showed that genotype and treadmill exercise had significant effects on the synaptic curvature both in the hippocampus (genotype: *F*_1,56_ = 17.4, *p* < 0.001; treadmill exercise: *F*_1,56_ = 5.0, *p* = 0.030; genotype × treadmill exercise interaction: *F*_1,56_ = 6.8, *p* = 0.012; Figure 3F) and prefrontal cortex (genotype: *F*_1,53_ = 5.1, *p* = 0.029; treadmill exercise: *F*_1,53_ = 5.3, *p* = 0.026; genotype × treadmill exercise interaction: *F*_1,53_ = 7.0, *p* = 0.011; Figure 3G). Tukey’s post hoc tests indicated that the synaptic curvature of the hippocampus (*p* < 0.001; Figure 3F) and prefrontal cortex (*p* = 0.001; Figure 3G) was significantly decreased in the 3×Tg-AD control group compared to the non-Tg control group. Treadmill exercise pretreatment increased the synaptic curvature both in the hippocampus (*p* = 0.001; Figure 3F) and prefrontal cortex (*p* < 0.001; Figure 3G) in 3×Tg-AD mice. Meanwhile, two-way ANOVA indicated that genotype and treadmill exercise had significant effects on the thickness of postsynaptic density, both in the hippocampus (genotype: *F*_1,56_ = 26.3, *p* < 0.001; treadmill exercise: *F*_1,56_ = 8.0, *p* = 0.007; genotype × treadmill exercise interaction: *F*_1,56_ = 4.1, *p* = 0.047; Figure 3H) and prefrontal cortex (genotype: *F*_1,55_ = 34.2, *p* < 0.001; treadmill exercise: *F*_1,55_ = 11.1, *p* = 0.002; genotype × treadmill exercise interaction: *F*_1,55_ = 4.9, *p* = 0.032; Figure 3I). Tukey’s post hoc tests indicated that the thickness of postsynaptic density of the hippocampus and prefrontal cortex was significantly decreased in the 3×Tg-AD control group compared to the non-Tg control group (*p* < 0.001; Figure 3F,I). Treadmill exercise pretreatment increased the thickness of postsynaptic density both in the hippocampus (*p* = 0.001; Figure 3F) and prefrontal cortex (*p* < 0.001; Figure 3I) in 3×Tg-AD mice.

### 3.3. Effects of Treadmill Exercise on the Expression of Synaptophysin (Syn) and PSD95 of the Hippocampus and Prefrontal Cortex in 3×Tg-AD Mice

**Figure 4:**
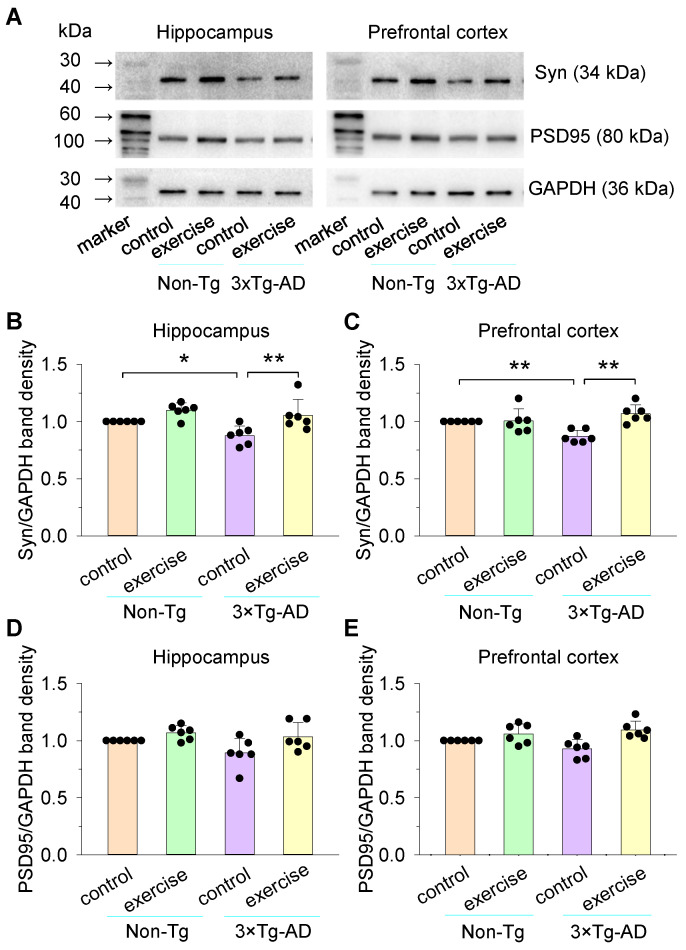
Treadmill exercise facilitates the expression of Syn and PSD95 of hippocampus and prefrontal cortex in 3×Tg-AD mice. (**A**) Representative western blots for Syn, PSD95, and GAPDH of hippocampus and prefrontal cortex homogenates were prepared from these four groups of mice. (**B**,**C**) Summarized data showed that Syn of hippocampus (**B**; * *p* < 0.05, n = 6) and prefrontal cortex (**C**; ** *p* < 0.01, n = 6) were significantly decreased in the 3×Tg-AD control group compared to the non-Tg control group and this decrease was blocked by treadmill exercise pretreatment both in the hippocampus (**B**) and prefrontal cortex (**C**) (** *p* < 0.01, n = 6). (**D**,**E**) There were no significant effects on the PSD95 between non-Tg control, non-Tg exercise, 3×Tg-AD control, and 3×Tg-AD exercise mice in the hippocampus (**D**; *p* > 0.05, n = 6) and prefrontal cortex (**E**; *p* > 0.05, n = 6). Immunoreactivity was normalized to GAPDH and presented as the percentage of the non-Tg control group. Each data set was obtained from 3 mice.

Syn is a specific presynaptic marker and PSD-95 is a specific postsynaptic marker for excitatory synapses [53,54]. We further investigated the expression of Syn and PSD95 of the hippocampus and prefrontal cortex in these four groups of mice (Figure 4A). For the hippocampus, two-way ANOVA indicated that genotype and treadmill exercise had significant main effects on the expression of Syn (genotype: *F*_1,23_ = 5.5, *p* = 0.029; treadmill exercise: *F*_1,23_ = 14.9, *p* < 0.001; Figure 4B), but there was not a significant interaction between genotype and treadmill exercise on the expression of Syn (genotype × treadmill exercise interaction: *F*_1,23_ = 1.2, *p* = 0.292; Figure 4D). For the prefrontal cortex, two-way ANOVA indicated that genotype had no significant main effect on the expression of Syn (genotype: *F*_1,23_ = 1.2, *p* = 0.278), but there was a significant interaction between genotype and treadmill exercise on the expression of Syn (treadmill exercise: *F*_1,23_ = 11.9, *p* = 0.002; genotype × treadmill exercise interaction: *F*_1,23_ = 11.2, *p* = 0.003; Figure 4C). Tukey’s post hoc tests indicated that the expression of Syn of the hippocampus (*p* = 0.025; Figure 4B) and prefrontal cortex (*p* = 0.005; Figure 4C) was significantly decreased in the 3×Tg-AD control group compared to the non-Tg control group. Treadmill exercise pretreatment increased the expression of Syn both in the hippocampus (*p* = 0.002; Figure 4B) and prefrontal cortex (*p* < 0.001; Figure 4C) in 3×Tg-AD mice. For PSD95 expression, two-way ANOVA revealed that genotype and treadmill exercise had no significant effects on the expression of PSD95 both in the hippocampus (genotype: *F*_1,23_ = 3.3, *p* = 0.082; treadmill exercise: *F*_1,23_ = 7.5, *p* = 0.013; genotype × treadmill exercise interaction: *F*_1,23_ = 0.9, *p* = 0.344; Figure 4D) and prefrontal cortex (genotype: *F*_1,23_ = 0.4, *p* = 0.514; treadmill exercise: *F*_1,23_ = 15.2, *p* < 0.001; genotype × treadmill exercise interaction: *F*_1,23_ = 3.3, *p* = 0.083; Figure 4E). The original Western blots are provided in Supplementary Figure S1.

### 3.4. Treadmill Exercise Enhanced the Axon Length and Dendritic Complexity of the Hippocampus and Prefrontal Cortex in 3×Tg-AD Mice

**Figure 5:**
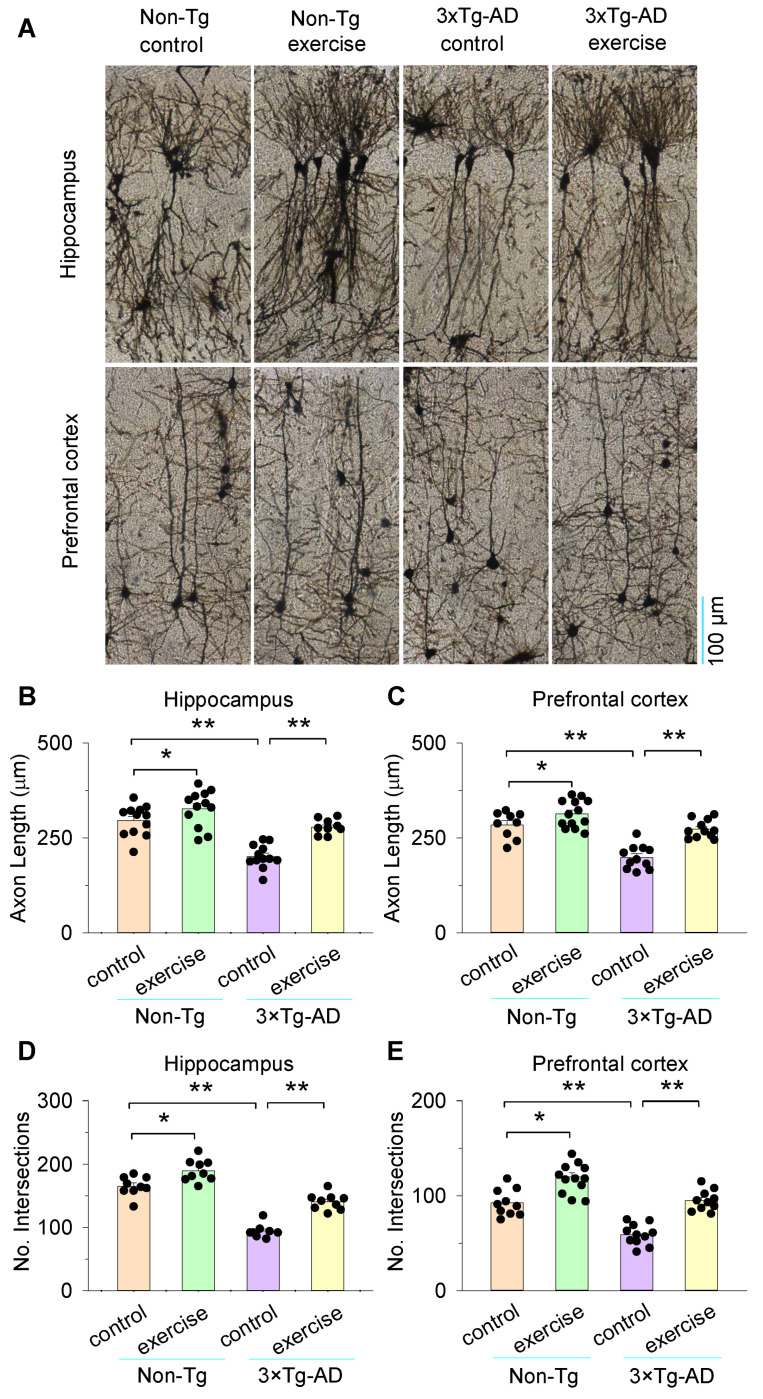
Treadmill exercise enhance the axon length and dendritic complexity of the Hippocampus and prefrontal cortex in 3×Tg-AD mice. (**A**) Representative Golgi staining images of hippocampus and prefrontal cortex in non-Tg control, non-Tg exercise, 3×Tg-AD control, and 3×Tg-AD exercise mice. (**B**,**C**) Axon length of hippocampus (**B**) and prefrontal cortex (**C**) were significantly decreased in the 3×Tg-AD control group compared to the non-Tg control group (** *p* < 0.01, n = 9–13 image sections, 3–5 cells/image section) and this decrease was blocked by treadmill exercise pretreatment both in the hippocampus (**B**) and prefrontal cortex (**C**) (** *p* < 0.01, n = 9–13). Treadmill exercise increased the axon length in the hippocampus (**B**) and prefrontal cortex (**C**) in non-Tg mice (* *p* < 0.05, n = 9–13 image sections). (**D**,**E**) Dendritic complexity of hippocampus (**D**) and prefrontal cortex (**E**) were significantly decreased in the 3×Tg-AD control group compared to the non-Tg control group (** *p* < 0.01, n = 9–13 image sections) and this decrease was blocked by treadmill exercise pretreatment both in the hippocampus (**D**) and prefrontal cortex (**E**) (** *p* < 0.01, n = 9–13). Treadmill exercise increased the dendritic complexity in the hippocampus (**D**) and prefrontal cortex (**E**) in non-Tg mice (* *p* < 0.05, n = 9–13 image sections). Each data set was obtained from 3 mice.

Axons and dendrites represent the structural basis of synaptic plasticity. We next assessed the axon length and dendritic complexity of the hippocampus and prefrontal cortex in 3×Tg-AD mice by Golgi staining (Figure 5A). Two-way ANOVA revealed that genotype and treadmill exercise had significant effects on the axon length of the hippocampus (genotype: *F*_1,45_ = 43.6, *p* < 0.001; treadmill exercise: *F*_1,45_ = 25.7, *p* < 0.001; genotype × treadmill exercise interaction: *F*_1,45_ = 4.1, *p* = 0.049; Figure 5B), the prefrontal cortex (genotype: *F*_1,43_ = 41.6, *p* < 0.001; treadmill exercise: *F*_1,43_ = 28.0, *p* < 0.001; genotype × treadmill exercise interaction: *F*_1,43_ = 5.4, *p* = 0.025; Figure 5C), and the dendritic complexity of the hippocampus (genotype: *F*_1,36_ = 166.8, *p* < 0.001; treadmill exercise: *F*_1,36_ = 57.5, *p* < 0.001; genotype × treadmill exercise interaction: *F*_1,36_ = 5.2, *p* = 0.029; Figure 5D) and the prefrontal cortex (genotype: *F*_1,42_ = 52.3, *p* < 0.001; treadmill exercise: *F*_1,42_ = 62.6, *p* < 0.001; genotype × treadmill exercise interaction: *F*_1,42_ = 5.7, *p* = 0.022; Figure 5E). Tukey’s post hoc tests indicated that the axon length and dendritic complexity of the hippocampus and prefrontal cortex were significantly decreased in the 3×Tg-AD control group compared to the non-Tg control group (*p* < 0.001; Figure 5B–E). Treadmill exercise increased the axon length and dendritic complexity in the hippocampus and prefrontal cortex in 3×Tg-AD mice (*p* < 0.001; Figure 5B–E). Meanwhile, treadmill exercise increased the axon length and dendritic complexity in the hippocampus and prefrontal cortex in non-Tg mice (*p* < 0.05; Figure 5B–E).

### 3.5. Treadmill Exercise Improved the Numbers of Dendritic Spines of the Hippocampus and Prefrontal Cortex in 3×Tg-AD Mice

**Figure 6:**
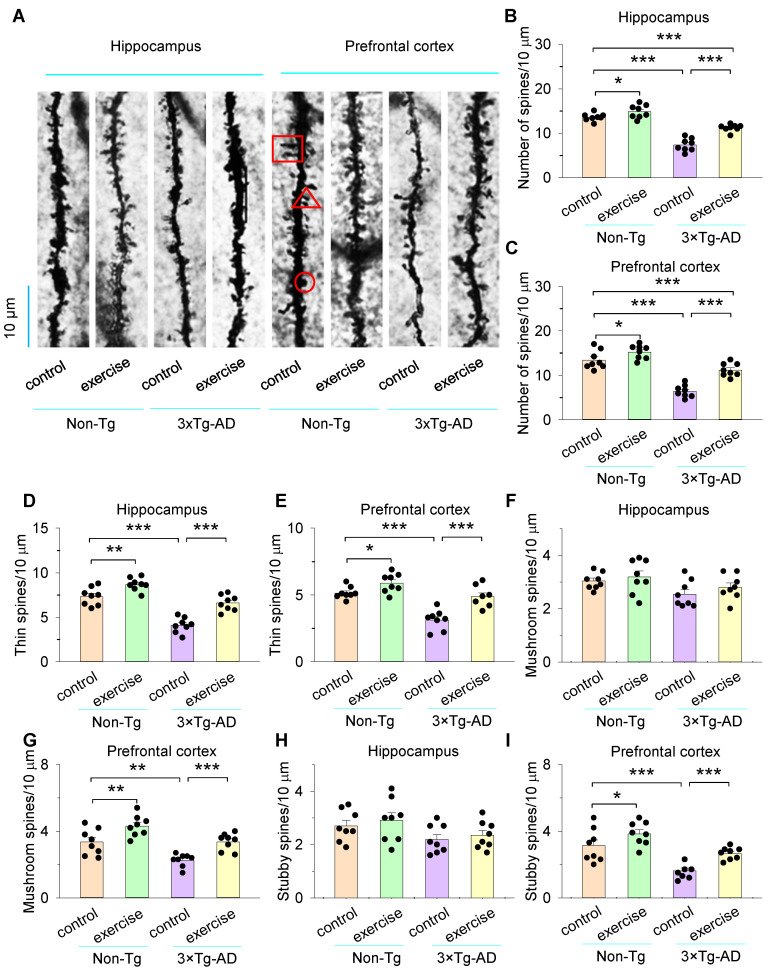
Treadmill exercise improves the dendritic spines numbers of hippocampus and prefrontal cortex in 3×Tg-AD mice. (**A**) Representative Golgi staining images of the secondary dendrites of the CA1 pyramidal neurons in the hippocampus and layer V pyramidal neurons prefrontal cortex in non-Tg control, non-Tg exercise, 3×Tg-AD control, and 3×Tg-AD exercise mice. The dendritic spines in rectangles, triangles, and circles are thin, mushroom, and stubby dendritic spines, respectively. (**B**,**C**) The spines numbers of hippocampus (**B**) and prefrontal cortex (**C**) were significantly decreased in the 3×Tg-AD control group compared to the non-Tg control group (*** *p* < 0.001, n = 8), and this decrease was blocked by treadmill exercise pretreatment both in the hippocampus (**B**) and prefrontal cortex (**C**) (*** *p* < 0.001, n = 8 dendrites, respectively). Treadmill exercise pretreatment increased the spines numbers of the hippocampus (**B**) and prefrontal cortex (**C**) in non-Tg mice (** *p* < 0.01, n = 8 dendrites, respectively). (**D**,**E**) The thin spines of the hippocampus (**D**) and prefrontal cortex (**E**) were significantly decreased in the 3×Tg-AD control group compared to the non-Tg control group (*** *p* < 0.001, n = 8), and this decrease was blocked by treadmill exercise pretreatment both in the hippocampus (**D**) and prefrontal cortex (**E**) (*** *p* < 0.001, n = 8). Treadmill exercise pretreatment increased the spines numbers of the hippocampus (**D**; ** *p* < 0.01, n = 8) and prefrontal cortex (**E**; * *p* < 0.05, n = 8) in non-Tg mice. (**F**–**I**) The mushrooms (** *p* < 0.01, n = 8), and stubby spines (*** *p* < 0.001, n = 8) of prefrontal cortex (**G**,**I**) were significantly decreased in the 3×Tg-AD control group compared to the non-Tg control group, and this decrease was blocked by treadmill exercise pretreatment both in the prefrontal cortex (*** *p* < 0.001, n = 8). Treadmill exercise pretreatment increased the mushrooms (** *p* < 0.01, n = 8) and stubby spines (* *p* < 0.05, n = 8) of the prefrontal cortex (**G**,**I**) in non-Tg mice. There were no significant effects on the mushrooms and stubby spines of the hippocampus between non-Tg control, non-Tg exercise, 3×Tg-AD control, and 3×Tg-AD exercise mice (**F**,**H**; *p* > 0.05, n = 8). Each data set was obtained from 3 mice.

Dendritic spines are the original sites of neuronal excitatory synaptic transmission, and their morphology and structure are dynamic both under normal conditions in vivo and under conditions of synaptic plasticity [55]. Previous studies have shown that the dynamics of dendritic spines is associated with learning and memory, while thin, mushroom, and stubby spines have a different role in learning and memory [56]. We further analyzed the dendritic spine numbers of the secondary dendrites and categorized them into three types (thin, mushroom, and stubby) based on the morphological characteristics of the spine head, neck, and length (see Section 2, Figure 6A). Two-way ANOVA revealed that genotype and treadmill exercise had significant effects on the numbers of spines both in the hippocampus (genotype: *F*_1,31_ = 140.0, *p* < 0.001; treadmill exercise: *F*_1,31_ = 36.8, *p* < 0.001; genotype × treadmill exercise interaction: *F*_1,31_ = 9.4, *p* = 0.005; Figure 6B) and prefrontal cortex (genotype: *F*_1,31_ = 89.4, *p* < 0.001; treadmill exercise: *F*_1,31_ = 31.5, *p* < 0.001; genotype × treadmill exercise interaction: *F*_1,31_ = 6.5, *p* = 0.017; Figure 6C). Tukey’s post hoc tests indicated that the numbers of spines of the hippocampus and prefrontal cortex were significantly decreased in the 3×Tg-AD control group compared to the non-Tg control group (*p* < 0.001; Figure 6B,C). Treadmill exercise pretreatment prevented the decrease in the number of spines both in the hippocampus and prefrontal cortex in 3×Tg-AD mice (*p* < 0.001; Figure 6B,C). Meanwhile, treadmill exercise increased the number of spines of the hippocampus (*p* = 0.043; Figure 6B) and prefrontal cortex (*p* = 0.039; Figure 6C) in non-Tg mice. Furthermore, the numbers of spines of the hippocampus and prefrontal cortex were significantly decreased in the 3×Tg-AD exercise group compared to the non-Tg control group (*p* < 0.001; Figure 6B,C).

Spines are considered thin if the length is greater than the neck diameter and the diameters of the head and neck are similar [47]. Thin spines emerge and disappear over a few days and concentrate biochemical signals such as Ca^2+^, providing the synaptic specificity required for learning [56,57]. Two-way ANOVA revealed that genotype and treadmill exercise had significant effects on the thin spines both in the hippocampus (genotype: *F*_1,31_ = 74.3, *p* < 0.001; treadmill exercise: *F*_1,31_ = 39.4, *p* < 0.001; genotype × treadmill exercise interaction: *F*_1,31_ = 3.5, *p* = 0.073; Figure 6D) and prefrontal cortex (genotype: *F*_1,31_ = 39.3, *p* < 0.001; treadmill exercise: *F*_1,31_ = 27.4, *p* < 0.001; genotype × treadmill exercise interaction: *F*_1,31_ = 4.4, *p* = 0.044; Figure 6E). Tukey’s post hoc tests indicated that the thin spines of the hippocampus and prefrontal cortex were significantly decreased in the 3×Tg-AD control group compared to the non-Tg control group (*p* < 0.001; Figure 6D,E). Treadmill exercise pretreatment blocked the decrease in the number of spines both in the hippocampus and prefrontal cortex in 3×Tg-AD mice (*p* < 0.001; Figure 6D,E). Meanwhile, treadmill exercise increased the number of spines of the hippocampus (*p* = 0.004; Figure 6D) and prefrontal cortex (*p* = 0.036; Figure 6E) in non-Tg mice.

Spines are classified as mushrooms if the diameter of the head is greater than the diameter of the neck [47]. Mushroom spines are the more stable and persist for months. They can be shifted from thin spines by long-term potentiation (LTP) [56,58]. Two-way ANOVA indicated that genotype and treadmill exercise had no significant effects on the mushroom spines in the hippocampus (genotype: *F*_1,31_ = 6.2, *p* = 0.019; treadmill exercise: *F*_1,31_ = 1.4, *p* = 0.245; genotype × treadmill exercise interaction: *F*_1,23_ = 0.06, *p* = 0.803; Figure 6F). For the prefrontal cortex, two-way ANOVA indicated that genotype and treadmill exercise had significant main effects on the number of mushroom spines (genotype: *F*_1,31_ = 23.3, *p* < 0.001; treadmill exercise: *F*_1,31_ = 24.4, *p* < 0.001 Figure 6G), but there was not a significant interaction between genotype and treadmill exercise on the number of mushroom spines (genotype × treadmill exercise interaction: *F*_1,31_ = 0.1, *p* = 0.723; Figure 6G). Tukey’s post hoc tests indicated that the number of mushroom spines was significantly decreased in the prefrontal cortex in the 3×Tg-AD control group compared to the non-Tg control group (*p* = 0.001; Figure 6G), and treadmill exercise increased the number of mushroom spines in the prefrontal cortex (*p* < 0.001; Figure 6G) in 3×Tg-AD mice. Meanwhile, treadmill exercise increased the mushroom spines (*p* = 0.003; Figure 6G) of the prefrontal cortex in non-Tg mice.

Spines are considered stubby if the length and width are equal [47]. Stubby spines are viewed as an immature type that is prevalent during early postnatal development and shows relative scarcity in the mature brain [59]. Stubby spines may participate in the homeostatic regulation of calcium and in the control of neuronal excitability [60]. Two-way ANOVA indicated that genotype and treadmill exercise had no significant effects on the stubby spines in the hippocampus (genotype: *F*_1,31_ = 5.8, *p* = 0.023; treadmill exercise: *F*_1,31_ = 0.6, *p* = 0.430; genotype × treadmill exercise interaction: *F*_1,31_ = 0.01, *p* = 0.810; Figure 6H). However, two-way ANOVA indicated that genotype and treadmill exercise had significant main effects on the number of stubby spines (genotype: *F*_1,31_ = 31.1, *p* < 0.001; treadmill exercise: *F*_1,31_ = 13.4, *p* = 0.001; Figure 6I), and there was not a significant interaction between genotype and treadmill exercise on the number of stubby spines (genotype × treadmill exercise interaction: *F*_1,31_ = 0.4, *p* = 0.522; Figure 6I) in the prefrontal cortex. Tukey’s post hoc tests indicated that the stubby spines of the prefrontal cortex were significantly decreased in the 3×Tg-AD control group compared to the non-Tg control group (*p* < 0.001; Figure 6I). Treadmill exercise increased the number of stubby spines in the prefrontal cortex (*p* = 0.005; Figure 6I) in 3×Tg-AD mice. Meanwhile, treadmill exercise increased the number of stubby spines (*p* = 0.042; Figure 6I) of the prefrontal cortex in non-Tg mice.

## 4. Discussion

Here, we have demonstrated that six-month-old 3×Tg-AD mice exhibited impaired spatial working memory, and treadmill exercise pretreatment prevented decline in spatial working memory in 3×Tg-AD mice. Treadmill exercise pretreatment led to increases in synapse numbers, synaptic structural parameters, the expression of Syn, the axon length, dendritic complexity, number of the dendritic spines, and restoration of structural synaptic plasticity of the hippocampus and prefrontal cortex in 3×Tg-AD mice. Meanwhile, treadmill exercise pretreatment enhanced synaptic plasticity by increasing synapse numbers, the axon length, dendritic complexity, and number of dendritic spines in the hippocampus and/or prefrontal cortex of non-Tg mice. Our findings suggest that exercise may serve as an effective intervention in the early stage to delay the progression of AD.

We found that the percentage of working memory errors was significantly increased in the 3×Tg-AD control group compared to the non-Tg control group, but no significant effects on the percentage of reference memory errors. Short-term memory and synaptic function loss are the initial and most common presenting signs of memory impairment and cognitive decline [35]. As the disease progresses, people gradually experience long-term memory loss leading to problems with multitasking and abstract thinking. Thus, this result suggests that six-month-old 3×Tg-AD mice are still in the early stages of AD progression. Moreover, 12 weeks of treadmill exercise pretreatment led to a significant decrease in the percentage of working memory errors on day 5 and day 6 of the acquisition session under the eight-arm radial maze test. The eight-arm radial maze is one of the most common paradigms to assess spatial working memory and spatial reference memory [61]. Spatial working memory is often used synonymously with short-term memory, but working memory allows for the manipulation of stored information, whereas short-term memory only refers to the short-term storage of information [62]. Spatial reference memory refers to long-term memories that are required for remembering information [63]. Furthermore, previous studies demonstrated that treadmill running reverses cognitive declines in 3×Tg-AD mice in the Morris water maze task, which tests hippocampal-dependent learning, including the acquisition of spatial memory and long-term spatial memory [64,65,66]. Taken together, six-month-old 3×Tg-AD mice exhibited diminished spatial working memory, and the robust decrease of working memory errors under the eight-arm radial maze test indicate that treadmill exercise pretreatments prevent decline in spatial learning and memory in the early stages of 3×Tg-AD mice.

We examined the potential mechanisms that might underline the treadmill exercise-induced decrease of working memory errors in six-month-old 3×Tg-AD mice. Structural synaptic plasticity is manifested as changes in the number and size of synapses, the length of synaptic active zone, the width of the synaptic cleft, synaptic curvature, and the thickness of the postsynaptic density [67]. It is believed that structural synaptic plasticity within the hippocampus and prefrontal cortex forms the cellular basis of learning and memory, which depends on different pre-and post-synaptic neuronal mechanisms [5,6]. We found that treadmill exercise pretreatments led to an increase in the synapse numbers both in the hippocampus and prefrontal cortex of six-month-old 3×Tg-AD mice. Meanwhile, treadmill exercise pretreatments increased the synapse numbers both in the hippocampus and prefrontal cortex in non-Tg mice. The length of synaptic active zone reflects the presynaptic neuronal mechanisms of structural synaptic plasticity, while the synaptic curvature and the thickness of the postsynaptic density reflect the postsynaptic neuronal mechanisms of structural synaptic plasticity [68,69]. The synaptic cleft is the structure responsible for the neurotransmitter transmission between presynaptic and postsynaptic neurons, and optimal shortening of the synaptic cleft may have an adaptive function of optimizing synaptic strength [37,38]. Indeed, we found that treadmill exercise pretreatments remarkably increased the length of the synaptic active zone, the synaptic curvature, and the thickness of the postsynaptic density, shortening the width of the synaptic cleft in the hippocampus and prefrontal cortex of six-month-old 3×Tg-AD mice. Syn is a marker protein of presynaptic vesicles of the nerve cells [70], while the PSD95 is a pivotal postsynaptic scaffolding protein that modulates the postsynaptic response to the presynaptic release of glutamate by regulating the anchoring of glutamate receptors to the PSD [71]. Previous studies have shown that Syn and PSD95 were downregulated in the cerebral cortex of seven-month-old 3×Tg-AD mice and were recovered by six months of voluntary exercise treatment [72]. Consistent with this study [72], we found that treadmill exercise facilitated the expression of Syn in the hippocampus and prefrontal cortex of six-month-old 3×Tg-AD mice. The Aβ and tau-induced disruption of synaptic function is also manifest as impaired LTP/LTD induction and network oscillations [37,73,74]. Meanwhile, Treadmill exercise decreases the APP, BACE-1, and Aβ burden in both the hippocampus and cortex in AD model mice [24,27]. It is thus likely that exercise-induced increases in the synapse numbers, synaptic structure, and the level of Syn lead to the enhanced efficacy of neurotransmitter release and prevention of the decline in spatial working memory of three×Tg-AD mice.

Axons, dendrites, and dendritic spines constitute the structural basis of synaptic plasticity. The axon is functionally specialized to transmit signals, whereas the dendrites are specialized to receive signals [75,76]. In vivo imaging indicated axonal abnormalities and dendritic breakage around amyloid plaques in a 4–12-month-old double transgenic APP/PS1 mouse model of AD and 3×Tg-AD mice [73,74]. In vitro studies using Aβ1–42 and oligomeric Aβ have revealed that 60 h Aβ treatment resulted in the degeneration of both the axons, neuronal somata, and neuronal network dynamics [77,78]. Previous studies have shown that treadmill running alleviated Aβ deposition and the level of tau in the hippocampus and cerebral cortex in 3×Tg-AD mice and high-fat diet-fed rats [66,67,79]. We found that the axon length and dendritic complexity of hippocampus and prefrontal cortex were significantly decreased in the 3×Tg-AD control group compared to the non-Tg control group. Treadmill exercise pretreatment increased the axon length and dendritic complexity in the hippocampus and prefrontal cortex in 3×Tg-AD mice. It is likely that treadmill exercise pretreatment maintains axon length and dendritic complexity in the hippocampus and prefrontal cortex in 3×Tg-AD mice. Meanwhile, treadmill exercise pretreatment increased the axon length and dendritic complexity in the hippocampus and prefrontal cortex in non-Tg mice, indicating that exercise induces an increased surface area to facilitate interactions with other neurons and leads to enhanced structural synaptic plasticity in non-Tg mice.

Dendritic spines increase the surface area for possible synaptic connections. Changes in the shape, size, and number of synaptic spines are thought to underlie memory formation and are observed in a variety of neurodegenerative diseases, including AD and Parkinson’s disease [80,81,82]. In vitro studies found that 48-h treatment with 0.5–1.0 μM Aβ1–42 reduced the dendritic spine/synapse density in hippocampal cultures up to a maximum of ~40% [83]. Here, we have shown that the number of total dendritic spines of the hippocampus and prefrontal cortex was significantly decreased in the 3×Tg-AD control group compared to the non-Tg control group. Treadmill exercise pretreatment blocked the decrease in the number of spines in the hippocampus and prefrontal cortex in 3×Tg-AD mice. However, the number of spines of the hippocampus and prefrontal cortex was significantly decreased in the 3×Tg-AD exercise group compared to the non-Tg control group, suggesting that 12 weeks of treadmill exercise pretreatment partially restore the loss of dendritic spines in six-month-old 3×Tg-AD mice. On the other hand, treadmill exercise increased the number of spines of the hippocampus and prefrontal cortex in non-Tg mice. Previous studies have shown that the dynamics of dendritic spines is associated with learning and memory, and thin, mushroom, and stubby spines have a different role in learning and memory [56]. Thin spines are more dynamic than mushroom spines, respond to synaptic activity, and are believed to be ‘‘learning spines’’, responsible for forming new memories during the synaptic plasticity process, accompanied by head enlargement [56,79]. Mushroom spines form strong synaptic connections, have the longest lifetime, and are therefore thought to be sites of long-term memory storage [56,79]. Stubby spines are viewed as an immature type that is prevalent during early postnatal development and show relative scarcity in the mature brain [59]. Indeed, we found that the thin spines (hippocampus and prefrontal cortex), mushroom spines (prefrontal cortex), and stubby spines (prefrontal cortex) were significantly decreased in the 3×Tg-AD control group compared to the non-Tg control group. This regional difference may be due to the time course of Aβ deposition, which initiates in the neocortex and progresses to the hippocampus in 3×Tg-AD mice [17]. Therefore, the dendritic spines of the neocortex are more severely impaired. Treadmill exercise pretreatment blocked the decrease in the number of thin spines, mushroom spines, and stubby spines both in the hippocampus and prefrontal cortex in 3×Tg-AD mice, while treadmill exercise increased the thin spines of the hippocampus and prefrontal cortex, mushroom spines of the prefrontal cortex, and stubby spines of the prefrontal cortex in non-Tg mice. A strong positive correlation between dendritic spine density in the hippocampus and memory has been demonstrated using the fear conditioning paradigm, Morris water maze, and object placement behavioral assessments [84]. It is likely that treadmill exercise pretreatment potentiates synaptic connections via an increase in dendritic spines. Such mechanisms might explain why treadmill exercise pretreatment prevents decline in spatial working memory in 3×Tg-AD mice. Exercise intervention is thought to be a safe and economic choice as a therapeutic or preventative strategy against several diseases. As such, exercise may serve as a promising preventive intervention to alter the progression of AD.

## Data Availability

The data presented in this study are available on request from the corresponding author.

## Supplementary Materials

The following supporting information can be downloaded at: https://www.mdpi.com/article/10.3390/cells11020244/s1, Figure S1: Full-length Western blots of the Syn and PSD95 expression data shown in Figure 4.
