# Supplementary material for: Treadmill Exercise Prevents Decline in Spatial Learning and Memory in 3×Tg-AD Mice through Enhancement of Structural Synaptic Plasticity of the Hippocampus and Prefrontal Cortex
Source: Cells. 2022 Jan 12;11(2):244. doi: 10.3390/cells11020244 (PMC8774241; doi:10.3390/cells11020244)
Supplement: Supplementary file 1 [file cells-11-00244-s001.zip › cells-1507194-SI.pdf]

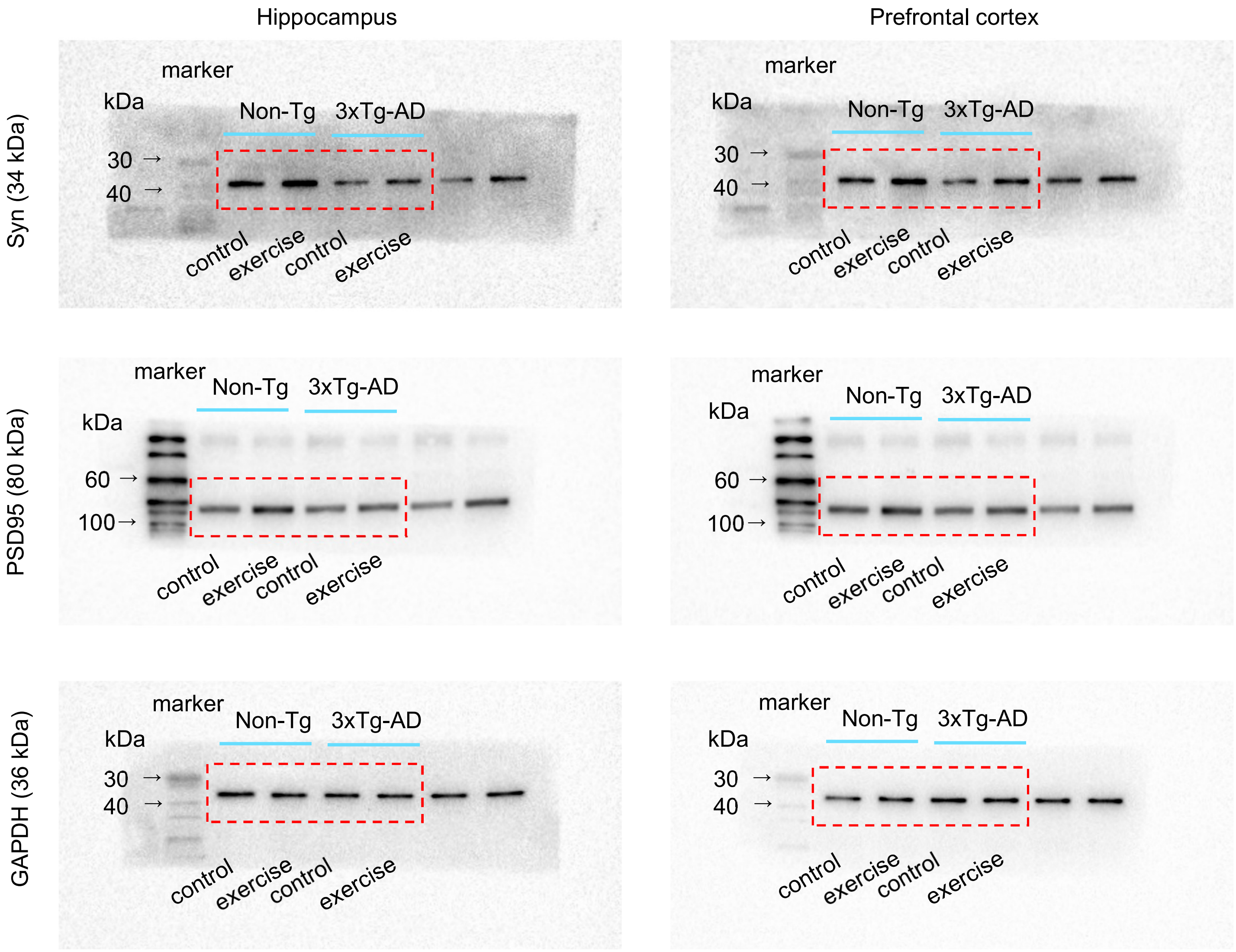


**Supplementary Figure S1. Full-length Western blots of the Syn and PSD95 expression data shown in Figure 4.** Red boxes indicate the bands that were cropped for the representative images shown in Figure 4.
